# Education and training of telemental health providers: a systematic review

**DOI:** 10.3389/fpubh.2024.1385532

**Published:** 2024-05-22

**Authors:** Qiaoling Jiang, Yongjia Deng, Jonathan Perle, Wanhong Zheng, Dilip Chandran, Jingru Chen, Feiyue Liu

**Affiliations:** ^1^Institute of Higher Education, Changsha University, Changsha, China; ^2^West Virginia University School of Medicine, Morgantown, WV, United States; ^3^Department of Behavioral Medicine and Psychiatry, West Virginia University School of Medicine, Morgantown, WV, United States; ^4^Mental Health & Counseling, Yale Health, New Haven, CT, United States; ^5^School of Economics and Management, Changsha University, Changsha, China

**Keywords:** telemental health (TMH), education and training, competency development, telepsychology, telepsychiatry, telebehavioral health (TBH)

## Abstract

**Objective:**

To conduct a systematic literature review of education and training (E&T) programs for telemental health (TMH) providers in the past 10 years to qualitatively clarify field offerings and methodologies, as well as identify areas for future growth.

**Methods:**

We searched five major electronic databases: PubMed, PsycINFO, Scopus, CINAHL, and Web of Science for original publications on TMH E&T from January 2013 to May 2023. We extracted information from each publication and summarized key features of training programs including setting, target group, study aims, training modality, methods of assessing quality, and outcomes.

**Results:**

A total of 20 articles were selected for the final review. Articles meeting inclusionary criteria were predominantly comprised of case studies and commentaries, focused on a TMH service/practice for a specific region/population, and were performed after 2020. All of the selected studies demonstrated a significant increase in the measured knowledge, skills, and abilities of the participants after TMH training. Nevertheless, there remains a lack of standardization of training methodologies, limited sample sizes and demographics, variability in study methodologies, and inconsistency of competency targets across studies.

**Conclusion:**

This systematic review highlighted the diversity of methods for TMH E&T. Future research on this topic could include more varied and larger-scale studies to further validate and extend current findings, as well as explore potential long-term effects of TMH training programs on both provider attitudes and patient outcomes.

## 1 Introduction

Telemental health (TMH) is used to refer to mental health services that are provided via telecommunications technologies (e.g., email, video, telephone, messaging programs). This field has demonstrated rapid development over the past three decades (1, 2). Advances in technology have enabled real-time distant consultations, assessments, therapy, and training opportunities. The distinction between traditional mental health providers and TMH lies in the mode of service delivery. Traditional mental health providers usually supply in-person services while TMH providers utilize telecommunication technologies for remote mental health service. While TMH continues to gain wider acceptance and adoption, driven by factors such as the need for increased access to mental health services, advancements in technology, and recognition of its efficacy and effectiveness (3), it has also faced criticism, especially on its use in psychotherapy and psychodynamic procedures (4, 5).

The COVID-19 pandemic further accelerated the implementation and adoption of TMH as an essential means of delivering mental healthcare. Many studies showed that when properly adapted from in-person practices, TMH approaches effectively promoted the mental health and well-being of participants with various mental problems (6). To ensure proper adaptation, a growing number of research studies detailed how TMH has been implemented, adopted, and perceived by diverse individuals and providers (7–9).

Many studies have demonstrated that education and training (E&T) in TMH is needed, as a lack of education can lead to suboptimal care and a lack of knowledge about how to address technological-related issues. Contrastingly, proper E&T has been suggested as positively impacting provider attitudes and knowledge, and subsequently patient outcomes (10–12). However, there is a lack of standardization in TMH E&T (13, 14). Some researchers have attempted to propose core competencies for TMH (12, 15–18) to include, but not necessarily be limited to, knowledge of the literature and evidence-based practices for different demographic characteristics, TMH modalities, and settings; evidence-informed methods of adapting in-person techniques (e.g., clinical work, interpersonal skills, rapport, communication) for digital administration; ethics of TMH practice; legality of TMH practice (e.g., cross-jurisdiction practice, data security); professionalism in practice, selection and troubleshooting of technology; diversity considerations of unique populations; administrative tasks (e.g., documentation, billing); and interdisciplinary collaboration. Nevertheless, to date, there has been little success in establishing standardized competencies, let alone efficient, scalable strategies for training existing TMH providers in said competencies. Additionally, very few E&T programs are dedicated to long-term career development for future TMH providers (19). For example, limited TMH education has been suggested as available in graduate education, affecting a provider’s knowledge and future use of TMH technologies in clinical care. This lack of training may be partially due to the relative youth of the field though has been suggested as an issue in need of addressing (20, 21).

To meet the increasing demand for evidence-based TMH providers, it is important to develop evidence-informed methods of E&T. The evidence-informed usage of TMH can guide ethical, legal, evidence-informed, and safe provider usage to not only maximize outcomes, but to reduce challenges that arise from the use of technology in clinical practice (22–24). Unfortunately, no known review has consolidated literature to clarify current field offerings and future directions. Therefore, there is a need for a systematic review concerning E&T of TMH providers to identify currently available programs, strengths and weaknesses of said programs, and the ongoing needs of the field. This information will help inform future adjustments in training design to better reflect the rapid changes in the field of TMH.

The objective of this study was to review and aggregate recent literature on TMH E&T to clarify field offerings and methodologies, as well as identify areas for further growth. Several research questions were derived to guide analyses: (a) who provided TMH E&T (i.e., setting), (b) who was the TMH E&T provided to, (c) what was the aim of TMH E&T, (d) how was the TMH E&T provided, (e) how was the quality of the TMH E&T assessed, and (f) what were the outcomes of the TMH E&T. Given limited prior literature to guide research questions, the current study was viewed as exploratory.

## 2 Methods

### 2.1 Study type

This study was conducted as a systematic review. The method of a systematic review was chosen over other methods, such as a scoping review, due to its rigorous and comprehensive approach in synthesizing existing literature to address focused research questions rather than summarize a broader and more diverse field of study.

### 2.2 Search strategy

**Figure 1:**
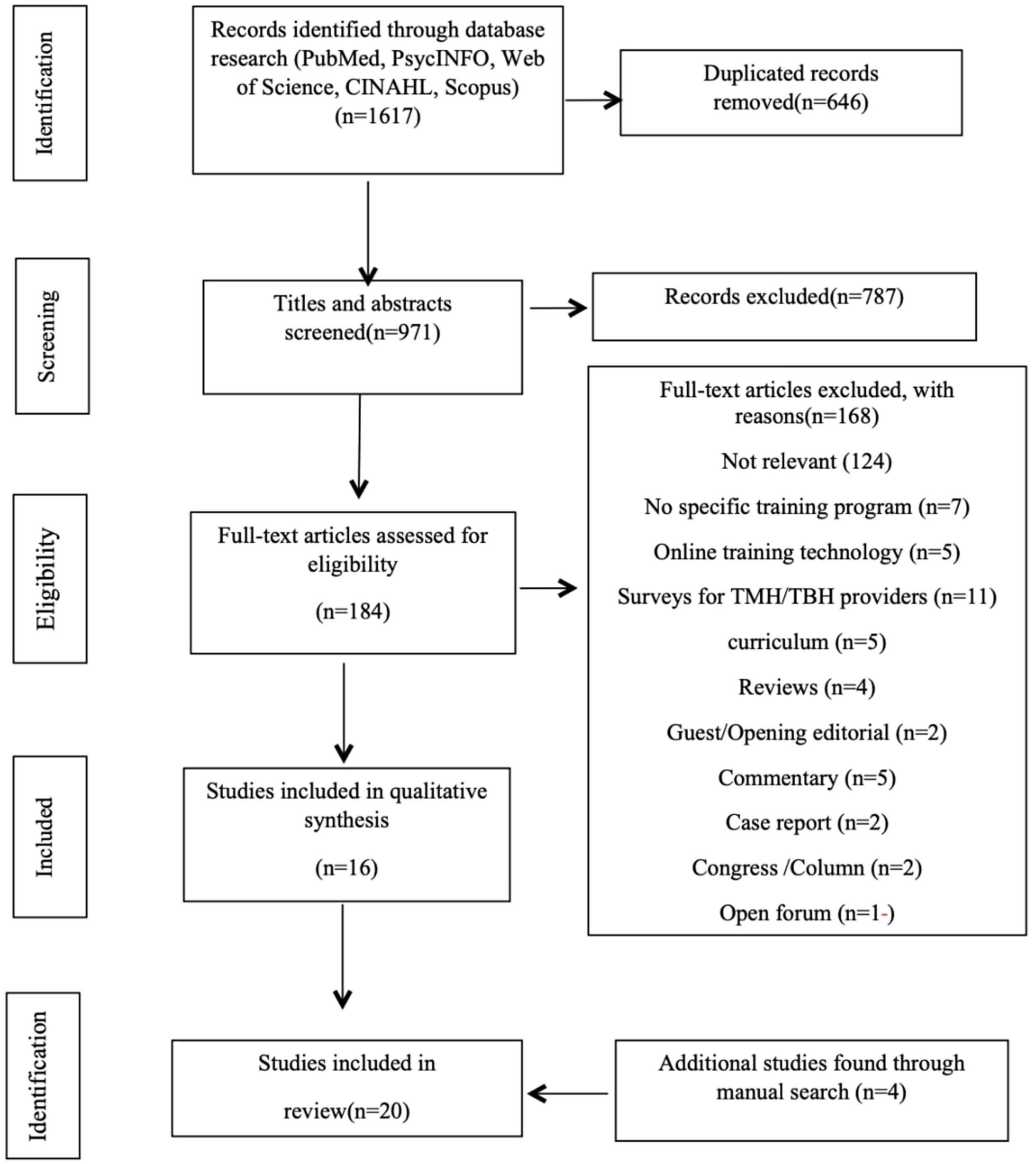
PRISMA diagram of search process.

This review was conducted according to the PRISMA standard with the involvement of every member of the research team (25). The screening process for determining which papers were to be analyzed in this study is depicted in Figure 1. We used the PubMed, PsycINFO, Scopus, CINAHL, and Web of Science databases to search TMH related English language articles published from January 2013 to May 2023. Keywords utilized were telemental health, TMH, telepsychology, telepsychiatry, telebehavioral health, TBH, and mobile mental health; all of these keywords were also separately paired with “education” and “training” to further expand the search. The full search strategies for each database, including complete keyword combinations, Boolean operator usage, and filtering criteria, can be found in the Supplementary material. Initially, title and abstract screening were conducted to assess relevance of the articles in the search results. Then, full-text screening was performed for eligible articles. Every article reviewed in this process was screened independently by at least two study members with any disagreements resolved through a group consensus. The Systemic Review Accelerator (SRA) was a computer tool that was utilized to facilitate this process.

### 2.3 Inclusion and exclusion criteria

The inclusion and exclusion criteria were based on relevance to education, training and competency development of TMH providers. Studies providing a description of education or training programs for mental health-focused telehealth providers were included. Contrastingly, articles focusing on only providers’ experiences or perspectives of TMH E&T were excluded. Papers that utilize remote or online technology to train providers in the fields of mental health or psychiatric informatics were also excluded, unless they were sufficient to cover all necessary training content and requirements of TMH itself. Papers examining the educational needs or the level of knowledge and skills of providers in TMH were also excluded if they were not in the context of a specific training program.

The current study elected to review the literature from only the past decade, as other reviews have covered July 2003 to March 2013 (3). Due to the rapid advances, a review of the last ten years is believed to better reflect the current status of TMH E&T than prior research.

### 2.4 Quality of studies

Each article meeting inclusionary criteria was assessed for relevance, quality, and contribution to the topic of TMH providers’ education, training, and competency development. The quality of studies was assessed using the Joanna Briggs Institute Qualitative Appraisal and Review Instrument (JBI QARI) Critical Appraisal checklist for Interpretive and Critical Research (Attachment). This quality assessment tool was modified for this systematic review; less relevant elements were removed as based upon consensus by the authors. The papers in this study were subsequently qualitatively analyzed through this tool to identify factors relevant to address research questions (See Tables 1, 2).

**Table 1:** Summation of articles included for review.

Authors	Setting	Target Group	Study Type & Objective	Training Modality	Training Assessment Measures	Results	Clinical Relevance	Limitations and Future Directions (As listed by original authors)
Mishkind, et al. (26)	In-person TMH training.	*N* = 36. Members of the U.S. Army Reserve that included psychologists, social workers, psychiatric nurse practitioners, occupational therapists, mental health specialists, medics, and other nonmedical support specialists.	A process improvement evaluation. Provide TMH knowledge and skills and to evaluate the effectiveness of the training program.	Half-day live session comprised of technical demonstration and troubleshooting, practical telehealth exercise, live/simulated clinical and technical scenarios.	Pre- to post-training assessment of TMH policy, procedures, and applications.	1.Surveys demonstrated successful preparation for military personnel to deliver TMH. 2.Training led to expansion of telehealth services by 40% during deployment. 3.Identification of several best practice recommendations: maintenance of in-person training, use of previous challenges to develop training scenarios, incorporation of training into daily activities, and tailoring of training while ensuring all trainees have the same knowledge base.	1.The program provided TMH training to a deploying military unit, to conduct a multifaceted evaluation of training and to make recommendations to improve training. 2.The training led to a significant expansion of TMH services throughout the southern region of Afghanistan, including direct provision of around 700 clinical encounters.	Limitations: a cause-and-effect relationship between the training and outcomes can be implied, but not confirmed. Future Directions: Training may include live sessions with technical demonstrations and troubleshooting overviews, practical telehealth exercises, and moderated after-action reviews.
McCord, et al. (27)	A non-profit Telehealth Counseling Clinic (TCC).	*N* = 25. Counseling Psychology doctoral students.	Case study of a telepsychology practicum training program. Focus on competencies for basic counseling, community, scientist-practitioner, and telepsychology.	Video recorded telepsychology sessions, didactic training, group supervision, tape review, and individual supervision.	Surveyed previous and current practicum students on experiences with the training program.	1.88% of respondents reported competence in ability to work with chronic mental health care issues. 2.75% agreed or strongly agreed that the program developed competence in working in desired settings.	1.Competencies can potentially serve as a model for psychology doctoral programs, practicum/extern placements, internships, and post-doctoral fellowships to increase attention on telepsychology training. 2. The program is valuable to both trainees and underserved communities.	Limitations: None listed by author. Future Directions: 1.Additional work is needed for developing agreed-upon telepsychology training standards. 2.Encouraged more development and implementation of telepsychology training programs. 3.Outcome and evaluation studies of telepsychology programs should increase, to evolve standards in telepsychology training.
Jefee-Bahloul et al. (28)	A global, humanitarian based TMH system that provides clinical consultations and supervision to health care providers in Syria.	*N* = undisclosed. Primary Care Centers or Mental Health Clinics.	A pilot/feasibility study. To describe a TMH network that uses a store-and-forward platform (asynchronous communication). Provide training and education for healthcare workers and clinical consultations in Syria (during a Civil War).	Overview of training for videoconferencing and technical navigation. Users uploaded relevant clinical information in writing or audio-video file. The assigned specialist asynchronously functioned as a resource for questions, conversations, and supervision.	Feedback from users to improve guidelines and referral process. Feedback used to refine procedural instructions and improve quality of clinical materials provided by the referring health care providers.	1.The majority of the pilot group users found the consultations to be clinically helpful, yet some had concerns about the scope of advice given. 2.The program was successful in allowing users to consult mental health providers for clinical purposes in a low-resource setting.	The study concluded that a global store-and-forward TMH system can effectively provide clinical supervision and training to healthcare providers to improve knowledge and skills in mental health assessment and treatment.	Limitations: 1. The efficacy of the TMH E and T materials was not analyzed in the study; the focus was on the logistical challenges (see below) of deployment. 2. TMH implementation in conflict zones/lower-income countries (poor bandwidth, time burdens, and the limited availability of supervisees). Future Directions 1.A sustainable and scalable program for trainees. 2.Partnerships with academic and non-governmental organizations to increase TMH clinical capacity and clinical consultations in low-income areas.
Alicata et al. (29)	Child and Adolescent Psychiatry TMH program at University of Hawaii.	*N* = undisclosed. Child & Adolescent Psychiatry Fellows (CAP).	Descriptive. Program aimed to train CAP fellows to provide behavioral health services through interactive video teleconferencing (IVTC) to rural communities.	IVTC/TMH service-learning curriculum, which integrated ACGME clinical competencies.	The ACGME Psychiatry Milestones to assess the trainee competency. Onsite Clinical Director and remote Program Director are responsible for supervision and evaluation.	1.Trainees provide direct services and consultations to patients on Hawaii Island and Maui County. 2.Collaboration with Mayo Clinic to examine the role of TMH in global service, training, education, and research.	The program integrated innovative technology in a successful evidence-based system of care treatment model and broadened access for psychiatric services for Hawaii’s youth.	Limitations. Hardware-based video teleconferencing systems are difficult to implement due to cost, immobility, technical expertise, internet requirements and lack of infrastructure/support in rural areas. Future Directions: The TMH program is examining different platforms and HIPAA-compliant cloud video conferencing for future usage.
Thew et al. (30)	Hong Kong health system effort to train therapists to adopt a British developed internet CBT training protocol.	*N* = 3. Therapists.	Mixed methods. To describe and evaluate a program of therapists training to deliver internet-based CBT for social anxiety disorder (CT-SAD).	After initial face-to-face case supervision, therapists received 7.5-day online iCT-SAD training. 3rd phase piloted the iCT-SAD with cases under weekly clinical supervision via Skype.	Assessment and Feedback from trainees regarding feasibility and acceptability of program and patient outcomes.	1.Increased therapist’s knowledge and skills of iCT-SAD. 2.All 6 patients treated by trainees completed treatment with improved outcomes and had sustained improvements at 3-momth follow-up visits.	The study concluded that this training improved the capability of Hong Kong-based therapists to deliver iCT-SAD and therefore showed substantial promise for expanding access to evidence-based treatment.	Limitations The number of trained therapists was small and the self-report assessment/skills tests have not yet been externally validated. Future Directions: Authors recommend a randomized controlled trial (RCT) to examine the efficacy of this intervention in patients and further research to develop, describe and evaluate therapist training procedures.
Caver et al. (31)	Veterans Affairs (VA) HealthCare System.	*N* = undisclosed. Psychology trainees and staff psychologists.	Descriptive. Overview of the VA’s TMH training program, to improve access to TMH by addressing barriers to training and implementation.	Web-based didactic courses, skills competency tests, supervision, and consultation in TMH for more advanced training.	In-person assessment plus telesupervision to provide qualitative assessments of trainees.	The study identified potential barriers to TMH training (lack of exposure, provider/patient preferences, provider social isolation and technical issues) and discussed solutions/strategies to address these barriers. Described actions at the VA Puget Sound Health Care System (VAPSHCS) as a model of training psychologists in the integration of TMH into clinical practice.	TMH provided effective mental health services to a wide range of patients and can potentially address barriers to accessing care. TMH may advance the field of mental health through continued research and development in the future, potentially serving as a model for other programs seeking to train psychologists for TMH.	Limitations: There were barriers at the level of the provider, patient, and system in the use of TMH. Future Directions: A need for further research on the feasibility and effectiveness of the clinical champion model for TMH innovation and training dissemination.
Perrin et al. (32)	Primary Care Psychology collaboration through a university.	*N* = >30. Psychology doctoral students across multiple primary care psychology training sites.	A commentary article. Presenting a case example of insights learned from the rapid deployment of telepsychology doctoral training/services during the COVID-19 pandemic.	Online training and transition to video-conferencing telepsychology services.	The development of a parallel virtual team meeting before each shift and increased supervisor time per trainee and case.	The study identified facilitators (trainee and supervisor resources, strong telepsychology training, and prior experience) and barriers to telepsychology deployment, as well as insights learned from the deployment process.	This study concluded that accelerated adoption of telepsychology services and doctor training amidst the COVID‐19 pandemic was crucial to maintaining and expanding mental health services at a critical time.	Limitations: Barriers included limited clinic capacity, scheduling, technology, accessibility, and diversity issues.
						These insights were divided into three categories: 1.Social stressors and issues that impede in-person visits. 2.Challenges of adapting provider training and supervision to a virtual model. 3.Adjusting to working with children and adolescents, as usual strategies for addressing disruptive behaviors no longer applied virtually.		Future Directions: continued research, evaluation, and policy development to advance the field of telepsychology and integrated primary care psychology training.
Wilkerson, et al. (33)	School of Social Work Educators in a large, North American public university.	*N* = 2,040. Social Workers practicing in a variety of clinical settings.	Descriptive. Describing how a School of Social Work rapidly deployed a free Continuing Education (CE) training program on the basics of TMH practice for the response to COVID-19, program’s usage, and user feedback.	Three online modules delivered using the Canvas Learning Management Platform.	Pre/ post program questionnaires were used to assess trainees. Learning platform analytics provided aggregated data on user-module page views and participation in learning activities. User feedback was collected through a CE program evaluation form.	The program was well-received by participants, with 98 out of 123 evaluation comments indicating appreciation for the program. Remaining comments had suggestions for content inclusion and technical improvements.	Helped provide social work educators/clinicians with necessary skills to deliver TMH services during the COVID-19 pandemic.	Limitations: Lack of a control group, long-term impact of the training was not assessed and potential for selection bias among participants. Future directions: Necessary to have a more rigorous evaluation of the effectiveness of TMH services and the development of best practices for TMH delivery.
Malhotra et al. (34)	Postgraduate training institute and 3 remote district hospital sites in India.	*N* = undisclosed. Psychologists, social workers, and technicians who were working with psychiatric patients.	Surveys, interviews, and clinical assessments. Assess feasibility & effectiveness of training non-specialists in telepsychiatric evaluations via video.	Video-based conferencing using Skype: training included didactic lectures, case discussions, role-plays, and hands-on training with the clinical decision support system (CDSS).	Surveys and interviews were conducted to evaluate trainee and trainer satisfaction. Clinical assessments evaluated the effectiveness of the training by comparing diagnostic agreement between trainees and trainers. A cost analysis was conducted.	1.Trainees were able to accurately evaluate patients for psychiatric conditions with their differential diagnoses concurring with those of the trainers 80–100% of the time, depending on diagnosis.	The study concluded that this program provides a feasible and cost-effective way to train non-specialist staff in diagnosing mental disorders.	Limitations: small sample size limits the generalizability of the findings; the study did not assess the long-term impact of the training on patient outcomes and poor internet connectivity and variable quality of the video/audio impeded training.
						2.All the trainees (6) and trainers (4) felt that the video-conferencing training was at least somewhat useful. 3.In-person training was around 150% more costly than video-conferencing training.		Future Directions: research should focus on larger sample sizes, longer-term follow-up, and assessment of the impact of training on patient outcomes.
Kroll, et al. (35)	Large midwestern Children’s Hospital.	*N* = 87 Mental and behavioral health providers.	Pre- and post-training survey. To examine the effectiveness of a TMH training initiative to rapidly train mental health providers to provide TMH in response to the COVID-19 pandemic.	Self-paced with 4 sets of training materials including pre-recorded videos, PowerPoint slides, and written materials.	Pre-training and post-training surveys.	1.The program helped providers become prepared to provide TMH services quickly and effectively with 43% of providers reporting confidence in providing TMH prior to training and nearly all (98%) after training. 2.The program helped providers appreciate the value of TMH in practice with 67% of providers reporting motivation to provide TMH services prior to training and 83% doing so after training.	TMH can be an effective way to provide mental health services during a pandemic. TMH training is instrumental in preparing providers to offer these services.	Limitations: Not all providers who accessed the training materials completed the pre-training or post-implementation surveys. Future Directions: No future directions as suggested by the author.
Olso et al. (36)	Federally-funded Mental Health Technology Transfer Center (MHTTC) in the Northwest USA.	*N* = 1,718 school mental health professionals. *N* = 309 parents and caregivers. Providing training and technical assistance (TA) to school mental health (SMH) professionals.	Descriptive. Presenting preliminary process and outcome data that compares the reach and impact of support before and during COVID-19-related restrictions.	Through in-person and online training, workshops, and coaching.	Surveys to assess the impact of both the in-person and online trainings.	Online training and technical assistance (TA) had a wider reach (2023 trainees vs. 1,254 participants) and a more diverse audience (higher proportion of non-white and female trainees) than in-person training, with no decrease in trainee satisfaction and perceived impact.	1.Training and TA for the implementation of evidence-based SMH interventions can be effectively delivered through an online format in the context of the COVID-19 pandemic.	Limitations: There was a low response rate for online events/follow-up surveys. There was no comparison/control group. Future direction: the MHTTC team will continue to refine and evaluate efforts, incorporating new measures of impact and seeking to identify which training formats are associated with the most positive outcomes.
							2.The program was beneficial to professionals and family members who support student mental health due to greater accessibility of training.	
Parish et al. (37)	Conducted in a primary care setting by the Department of Psychiatry and Behavioral Sciences and Public Health Sciences at the University of California, Davis, United States.	*N* = 5. Mental health clinicians who were providing asynchronous telepsychiatry (ATP).	A clinical outcome-based study. To evaluate and define an ATP training model for primary care physician (PCP)-integrated behavioral health clinicians.	Clinical and procedural training through seminars, case supervision, and case discussions. For patient encounters, a video-recorded session was sent electronically to a consulting psychiatrist. Providers were also able to consult each other through electronic medical record messaging or via phone.	The training needs for ATP clinicians were assessed through supervision by telepsychiatry & expert feedback sessions conducted at various time points during the study.	1.Recommended skillsets for ATP interviewers: (1) comprehensive skills in brief psychiatric interviewing (2) adequate general knowledge base in behavioral health conditions and therapeutic techniques and (3) clinical documentation, integrated care/consultation practices, and e-competency skill sets. 2.Technology training recommendations include: (1) awareness of privacy/confidentiality for electronic data gathering, storage, management, and sharing; (2) technology troubleshooting; and (3) video filming/ retrieval.	1.This training program provides a model for delivering mental health care to patients in primary care settings through ATP. 2.This suggested model of training for ATP interviewing skills could be used for PCP-integrated behavioral health clinicians.	Limitations: 1.There was a small sample size. 2.Training methods were only examined for behavioral health clinicians only (no nurses for example). 3.There was no assessment of necessary training dose (e.g., time required for training). 4There was no objective rating of expert feedback. Future Directions: more rigorous studies of technology-focused, behavioral health services are needed.
Felker et al. (38)	The VA Puget Sound Health Care System (VAPSHCS).	*N* = 100. Interdisciplinary mental health providers including psychologists, social workers, psychiatrists, nurses, and others not specified.	Evaluation of program development and implementation. To assess the effectiveness of the program and implementation with interdisciplinary mental health providers in the VA system.	A mixed-methods approach: 8-h workshop specific to the practical aspects of providing TMH, hands-on training using the TMH equipment, and a VA-required TMH skills assessment.	The RE-AIM Framework assessed reach, effectiveness, adoption, implementation, and maintenance.	1.Providers reported satisfaction with the training program (95%). 2.The training significantly increased providers’ perceptions of their TMH knowledge, skills, and interest in TMH. 3.The number of providers using TMH and patients who received TMH nearly doubled; 16% of patients received services from providers who had participated in the training and 39% of providers provided TMH services compared to 16% prior. 4.Provided insights into barriers to the use of TMH for providers with no prior exposure and with prior exposure to TMH.	1.Increased access to care for patients who may have difficulty accessing traditional in-person mental health services, resulting in a doubling in the number of patients receiving such services after implementation. 2.The program helped to address some barriers to the use of TMH, such as concerns about patient privacy and confidentiality.	Limitations: there was a lack of a control group to compare TMH implementation without training. Future Directions: research should evaluate the long-term sustainability of the training program and its impact on patient outcomes, as well as the use of TMH in other clinical populations and settings.
McCord et al. (39)	Texas A&M Tele-Behavioral Care Program (TBC).	All psychology trainees in the system.	Case study. To provide guidance and recommendations for mental health training programs during public health crises (including COVID-19).	Transitioned from a hub and spoke model. Ensuring trainee support by utilizing a tele-supervision model, providing emergency consultation and boundary setting solutions.	Anecdotal reports and observations of trainees were used to assess the training program.	The majority of trainees and patients experienced no significant differences in their care and training; a minority of both either struggled to adapt to novel changes or thrived.	The study provided solutions to common pitfalls in Telepsych training (communications, logistics, training, emotional support for patients, and boundary setting).	Limitations: regarding telehealth service delivery include the potential for technical difficulties, privacy and security concerns, and the need for specialized training.
							The study also can inform implementation in other sites, which is clinically relevant for mental health training programs during public health crises.	Future directions: development of telehealth-specific competencies, the integration of telehealth into existing training programs, and the use of technology to enhance training and supervision.
Dopp et al. (40)	Health service psychology training programs at two universities.	*N* = 19. Clinical or counseling psychology students, with some experience with telepsychology training.	Mixed methods. To investigate students’ perceptions of competence in telepsychology domains and their perspectives in telepsychology training.	Video conferencing, telephone/audio calls, text messages, e-mails, mobile applications, and Web/Internet-based programs.	Quantitative and qualitative data using the Telepsychology Competency Rating Scale (TCRS) and interviews were used to assess the training experience and telepsychology competencies for trainees.	Identification of 12 qualitative themes of participant responses: five helpful aspects; five challenges; and two themes about internship.	The study informed telehealth training expansion efforts through an analysis of telepsychology training experiences.	Limitations: 1. Small sample size limited the generalizability of findings. 2.A disparity between the roles of the participants and research team, resulting in each side failing to understand the other’s perspectives. 3.Doctoral students who are not “early adopters” might have different training needs and challenges. Future directions: These should focus on how to best define, measure, and promote competency development in this emerging specialty area.
Atuel et al. (41)	Master of Social Work program at a large university.	*N* = 136. 61 in peer-to-peer role play (RP) group and 75 in virtual client-trainer (VC-T) group. Graduate level social work students.	Quasi-experimental nonequivalent groups study. To compare the effectiveness of a standardized peer-to-peer RP and a VC-T in training graduate-level students in the development of interviewing and clinical skills related to working with the military population.	Clinical training utilizing either RP or the VC-T.	Pre-test and Post-test clinical skills assessment, self-report online survey and clinical skill assessment using the Military Objective Structured Clinical Examination Scale (MOSCE).	Both RP and the VC-T were equally effective in improving clinical skills and competencies, specifically in the areas of initial engagement, recognition, and response to symptoms of PTSD and suicide, military cultural competence, and overall competence.	The study concluded that VC-T is a training tool that has greater reach compared with RP, but is potentially just as effective.	Limitations: 1. There was a small amount of training time and the use of self-report data. 2.There was no control group. 3.The study did not assess the long-term effects or sustainability of the training interventions. Future Directions: no future directions suggested by the author.
Merrill, et al. (17)	N/A.	N/A.	Commentary article. To provide evidence- and consensus-based, interprofessional TMH competency framework to the field of social work.	N/A.	N/A.	The Coalition for Technology in Behavioral Science interprofessional TMH competencies framework can be a valuable guide for Licensed Clinical Social Workers (LCSWs) who have begun using TMH service delivery for psychotherapy services.	This commentary concluded that the TMH competencies support existing clinical practices, as intended for TMH trainees and practitioners. The framework can be used to integrate clinical social work professional development, research, and training.	Limitations: 1. Developed in 2017 and may not fully reflect the current state of TMH practice. 2.Focused on LCSW’s in the USA, and the competencies may not be applicable to other countries or cultures. Future directions: The need for ongoing research to evaluate the effectiveness of TMH services and the development of additional competencies to address emerging issues in TMH practice.
Buck et al. (42)	Community-based behavioral agencies (the setting of training is not clearly mentioned).	*N* = undisclosed. mHealth support specialists (mHSS).	Commentary article. To describe key principles to guide digital mental health training and provide examples from the FOCUS mHealth effort.	A virtual training program over 2 weeks, subsequent website access to all training materials and ongoing support through consultation calls.	Not mentioned.	The Simple, Accessible, Inverted, Live (SAIL) model was anecdotally able to facilitate successful and rapid training of an mHealth workforce.	This study concluded that the SAIL model has the potential to remove key obstacles to the implementation and dissemination of digital health interventions for mental health.	Limitations: 1. This was a descriptive study; the efficacy of the SAIL model has yet to be experimentally verified. 2.The program detailed in this paper was limited to mHealth support roles. Future Directions: long-term cost and sustainability of this program are not known.
Felker et al. (43)	Developed by the Behavioral Health Institute (BHI) at the University of Washington.	N = undisclosed. Community mental health clinics, providers, and administrators who had limited TMH knowledge.	Commentary article. To describe how evidence-based implementation strategies were used to develop a framework to create and implement a TMH training program.	BHI developed and delivered a series of TMH training sessions, which included both webinars and online courses, the Plan-Do-Study-Act (PDSA) cycle approach, and a traditional didactic lecture format over Zoom.	The RE-AIM Framework was used to structure outcomes and evaluate the value, feasibility, and sustainability of ongoing TMH training.	The program reached many participants (At least 2,655 unique learners attending webinars and nearly 6,800 unique learners taking online courses as of June 2022).	The study concluded that the training program provided relevant and effective TMH training to behavioral health professionals to improve access to care for individuals in need of mental health services during the pandemic and beyond.	Limitations: the study was unable to assess how many providers and administrators changed their behaviors, and developed confidence or competence.
						Participants (81.9%) rated the training as relevant, useful, and impactful, and many reported increased confidences in using telemental health technologies to provide care.		Future Directions: work is needed to improve the website, outreach, and communication efforts.
Tyagi et al. (44)	Primary care settings in rural India.	*N* = 23 Female community health workers through the Accredited Social Health Activist (ASHA) Programme.	Acceptability study Review a digital program for training community health workers (CHW’s) in the detection and referral of patients with schizophrenia in community settings in rural India.	A digital training prototype, consisting of a series of short videos, PowerPoint slides with narration, images, and graphics, as well as links to relevant resources and materials.	Focus group discussions among trainees were used to assess the acceptability of digital training programs. Documented feedback from ASHA trainees.	Participants offered positive feedback about the training content and reported a positive experience with digital learning. During the focus group discussions, three themes were repeatedly mentioned: Recognizing schizophrenia, understanding symptoms and impact of stigma.	The study concluded that the digital training program has the potential to equip ASHA’s with the necessary knowledge and skills to improve the detection and referral of schizophrenia in rural India, which could lead to improved care and outcomes.	Limitations: 1. The sample size was small. 2.The program was only tested in one region; there is a need to be tested in different settings for greater generalizability; possibility for some further region-specific modifications if necessary to respond to diverse languages, cultures, and contexts. Future Directions: pilot test the digital training content with a larger cohort.

**Table 2:** Joanna Briggs Institute Qualitative Appraisal and Review Instrument (JBI QARI) critical appraisal checklist for interpretive and critical research.

Criteria Yes (1) Not clear (0) No (−1)
Research question or aim: is the research question or aim clearly stated? Methodological congruence: is the methodology congruent with the research question and approach? Data collection: do the data collection methods align with the research question and approach? Ethics: is there evidence of ethical considerations in the research? Findings: are the findings adequately discussed in the context of existing literature? Transferability: are there enough details to judge if the findings could apply to other contexts? Reflexivity: is the influence of the researcher on the research process, findings, and limitations acknowledged? Overall appraisal: does the study address the research question adequately?
Overall appraisal: does the study address the research question adequately?

## 3 Results

### 3.1 Review composition

The electronic searches identified 971 articles. An additional 4 were identified by a manual search from references. The abstracts of all 971 papers were reviewed with 184 meeting the inclusion criteria and 787 excluded. Based on full-text reading of the remaining 184, a further 168 papers were excluded, adding 4 additional records searching for manually and leaving a final set of 20 studies for inclusion in this review (Figure 1).

### 3.2 Characteristics of studies

A summary of the remaining 20 study’s objective, target group, training modality, outcomes and other characteristics can be seen in Table 3.

**Table 3:** Assessment of the quality of the 20 studies.

Study	C1	C2	C3	C4	C5	C6	C7	C8	Total
Mishkind et al. (26)	1	1	1	1	1	1	1	1	8
McCord et al. (27)	1	1	0	1	1	0	0	1	5
Jefee-Bahloul et al. (28)	0	1	1	1	1	1	0	1	6
Alicata et al. (29)	1	1	1	1	1	1	0	0	4
Thew et al. (30)	1	1	1	1	0	0	0	1	5
Caver et al. (31)	0	0	0	0	1	1	0	0	2
Perrin et al. (32)	1	1	1	0	1	1	0	0	5
Wilkerson et al. (33)	1	1	1	1	1	1	1	1	8
Malhotra et al. (34)	1	1	1	1	1	1	1	1	8
Kroll et al. (35)	1	1	1	1	0	1	0	1	6
Olso et al. (36)	1	1	1	0	1	1	0	1	6
Parish et al. (37)	1	1	0	1	1	0	1	1	6
Felker et al. (38)	1	1	1	1	1	1	1	1	8
McCord et al. (39)	1	1	0	0	1	1	1	1	6
Dopp et al. (40)	1	1	1	1	1	1	1	1	8
Atuel et al. (41)	1	1	1	1	1	1	0	1	7
Merrill et al. (17)	1	1	1	1	1	1	1	1	8
Buck et al. (42)	1	1	0	1	1	1	1	1	7
Felker et al. (43)	0	0	1	1	0	1	0	0	3
Tyagi et al. (44)	1	1	1	1	1	1	0	1	7
Total	17	18	15	16	17	17	6	16	6.16

According to the quality score, 6 studies were rated as 8, and 2 as 3,2, which are descriptive studies. The average score is 6.15, which indicates that the overall quality is high. Out of 20 studies, 16 studies have clearly stated the research question or aim about the TMH E&T, because 3 studies are not a research study with a specific research question or aim (28, 31, 43) and also 2 studies do not explicitly state the research approach for the TMH E&T program (30, 43). Instead, they provide an overview of the TMH E&T program (30) or focus on describing the methodology and outcomes of the program (17, 27). The data collection methods of 15 studies align with the research question and approach, and the other 5 studies do not provide detailed information on the specific data collection methods used (27, 31, 37, 39, 42). There is evidence of ethical considerations in 16 studies, while 4 studies do not provide detailed information on the ethical considerations of the research itself (31, 32, 36, 39), they do acknowledge the importance of ethical guidelines and considerations in the provision of telepsychology services. In terms of findings and transferability, 17 studies adequately discussed the findings in the context of existing literature, and there are enough details to judge if the findings could apply to other contexts. However, out of 20 papers, only 6 studies acknowledged the influence of the researcher on the research process, findings, and limitations, while most of the studies did not. 16 studies addressed the research question adequately.

Despite the analysis spanning ten years, review suggested that a majority (i.e., *n* = 14, 70%) of articles meeting inclusionary criteria were published following 2020.

### 3.3 Providers of TMH E&T

The TMH training programs were held in various settings, including, but not limited to university counseling centers, community clinics, private practices, VA hospitals, and prisons/jails. A significant portion of the settings were affiliated with academic institutions. Many clinical service settings, such as VA, expanded TMH E&T during the COVID-19 pandemic due to the urgent need for TMH at that time. While beneficial to all patient demographics, the option for TMH was especially useful for certain populations, such as children and adolescents (29), people with social anxiety disorder (30), and individual experiencing schizophrenia (44).

### 3.4 Users of TMH E&T

The target trainees were diverse and included military personnel, doctoral students, healthcare providers, interdisciplinary practitioners, and a mix of students and qualified professionals. The majority, however, were psychology/social worker students or clinicians. Of note, each program had a training focus area and goals unique to the training program.

### 3.5 Aims of TMH E&T

Almost all training programs included in the study focused on enhancing the skills and knowledge of TMH providers. Study objectives collectively aimed to improve the reach of mental health professionals, especially in the context of public health crises and underserved communities, while leveraging technology for future training and service delivery. While some studies described specific training activities among specific trainee populations (e.g., psychology practicum training for specific interventions, telepsychiatry) (27, 37), others assessed the feasibility and efficacy of TMH E&T more broadly among different cultural (30), and clinical settings (34). A few studies focused on promoting access to mental health services through telehealth, addressing barriers (31), and examining participant experiences with online training tools (35). Additionally, some studies focused on comparing different training modalities, and exploring factors influencing telepsychology training from the perspective of doctoral students (40).

### 3.6 Methods of providing TMH E&T

**Figure 2:**
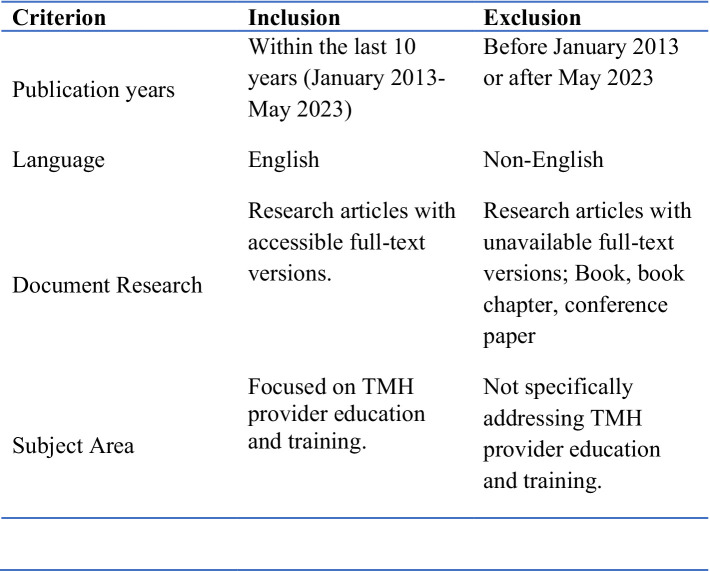
Inclusion and exclusion criteria.

While the training modalities all included virtual components such as web-based courses, live sessions or asynchronous audio-video communications, there were some notable differences. For example, some programs incorporated specialized in-person training (36), or diverse formats (e.g., training to use a specific online platform) to meet the specific needs of the training (37). A mixed-methods approach, combining various training components, was evident in some studies to provide a comprehensive learning experience (38). Many training programs involved interactive elements such as group supervision, role-plays, case discussions, and hands-on training to engage participants (34, 37, 39) which were delivered via digital methods including videoconferencing, online platforms, and other digital resources (See Figure 2).

### 3.7 Quality assessment of TMH E&T

Most evaluations of the effectiveness, feasibility, and impact of TMH training programs were done through surveys. Those included competency-based evaluations (29), feedback (34, 37), programmatic evaluations (28, 33), and sustainability assessments (43). Most assessments focused on using feedback and data to improve training programs, guidelines, and processes (36, 40, 43); however, some assessments, particularly in programmatic evaluations, aimed to provide resource savings in training delivery (42). One study used both quantitative and qualitative data to evaluate the effectiveness of training programs (40).

### 3.8 Outcomes of TMH E&T

All training programs were found to be effective in achieving their goals, including expanding access to services, improvement of competencies, facilitating global collaboration among international institutions, and facilitating rapid adaptation during time-sensitive public health crises. User satisfaction was a common theme, indicating that participants found the training programs to be helpful, timely, and relevant (34, 36, 38). The results also highlighted the adaptability of these programs in response to challenges, such as the rapid adoption of telepsychology during the COVID-19 pandemic, and the importance of collaboration, outreach, and ongoing support in reaching diverse audiences and expanding the positive impact of TMH training efforts (29).

## 4 Discussion

The current systematic review of TMH E&T summarized the setting, target group, study aims, training modality, quality of training assessment, and outcomes of studies over the last 10 years.

While present to some degree prior, the study of TMH E&T demonstrated the most notable growth since 2020. While definitive reasons for this finding are unclear, as they were often not directly addressed in each study’s methodology, it is hypothesized that the growing recognition of the importance of TMH E&T arose in response to the exponential increase in clinical usage following the advent of COVID-19 (i.e., providers experienced a rapid and unanticipated transition to telehealth, despite having limited prior training). As a result, providers, training programs, and researchers recognized the gap in both literature and training related to TMH competencies, prompting the increase in study.

### 4.1 Current field offerings

Analysis suggested that while still in its relative infancy, literature on TMH E&T is developing. Data indicated that trainings have occurred across a wide variety of locations (e.g., university counseling centers, community clinics, hospitals), and for healthcare providers across professional developmental levels (e.g., doctoral level, licensed providers). While professional emphasis varied, the majority of identified literature focused on psychology or social worker students and licensed professionals rather than other healthcare specialties. Related to aims, training programs predominantly focused on developing both knowledge and skill domains, two equally important, but different, components necessary to develop competency in the numerous telehealth competencies (e.g., ethics, legal, adaptations of practice) (45). Such programs included E&T through practicum training and focused continuing education provided in-person, via technology (e.g., web-based courses, live sessions), or a hybrid approach of both. Trainings were broadly concluded as feasible, and yielding positive impact on provider self-perceived knowledge of TMH, as well as allowing for expansion of access to clinical services. Finally, satisfaction of the trainings was generally high among trainees, suggesting the sustainability of the outlined programs either as currently designed, or as adapted/updated for future use.

### 4.2 Key factors and themes

As based upon primary findings, key factors and themes for the studies can be summarized as follows:

- Supervision and manpower: availability of flexible on-ground supervisors is crucial for effective TMH E&T. This includes ensuring there is adequate support, follow-up, and ongoing assistance for trainees (28, 39).
- Technology quality and reliability: the success of TMH interventions is contingent upon the quality and reliability of the technology used in training. Factors such as audiovisual communication quality and internet signal strength play significant roles (34, 38, 39, 42).
- Cultural and linguistic appropriateness: ensuring interventions are culturally and linguistically appropriate is highlighted as an important factor for successful TMH E&T (28).
- Integration into healthcare systems: successful integration into existing healthcare systems is crucial for the success of TMH interventions. Addressing broader social and political factors affecting mental healthcare is also emphasized (28).
- Addressing barriers: barriers at provider, system, and patient levels, such as lack of familiarity with technology, limited access, concerns about quality, and lack of a supportive, training-oriented environment must be addressed through comprehensive training programs (31, 40).
- Continuous interaction and feedback: constant interaction, ongoing support, and a phased approach to training are essential to ensure effectiveness (34, 39, 42).
- Choice of applications and training approach: factors like the choice of applications, clinician engagement, and the overall training approach significantly impact the effectiveness of TMH E&T (38, 43).
- Evaluation methodology: the use of frameworks like PARiHS and RE-AIM for evaluation is emphasized, underlining the importance of robust evaluation methodologies (38, 43).
- These factors collectively stress the need for a comprehensive, context-specific, and culturally sensitive approach to ensure the effectiveness of TMH E&T programs.

### 4.3 Limitations of the literature and areas for future TMH E&T growth

Reviewed studies collectively had limitations that may affect their generalizability. First, a majority of studies were case examples of local/regional training programs. While serving as a template for other locations, direct application may not be possible to other settings/locations without adaptation. Similarly, most existing studies were based on small sample sizes and specific target groups, and therefore limit the generalizability of the findings. Larger-scale research in more diverse settings is needed to confirm the generalizability of the results. Additionally, many studies relied on one-time self-reported surveys as opposed to more objective data collection of specific operationally-defined targets, such as changes in attitude towards TMH, usage of TMH, how providers adapted (or did not adapt) practices following TMH E&T, locations served via TMH, and long-term use of TMH. Objective evaluation is believed necessary, as just because a provider gains new knowledge and skills does not necessarily mean that they will integrate such information into their practice in the immediate or long-term, especially if barriers present (e.g., lack of time, financial challenges). As a result, external validation of assessments currently utilized, as well as longitudinal data collection are required (30). Related to the training itself, there remains a lack of uniformity in terms of how the information is presented (e.g., length, methodology) (27). More concerning, many studies failed to define what was actually being taught in terms of TMH modality (e.g., video vs. email) or competencies. Finally, a wider range of provider demographic characteristics is needed to determine the influence of location (i.e., rural, urban), race, ethnicity, socioeconomic status, treated population, and technology modalities (e.g., video, telephone, email) on training outcomes. Towards this end, additional evaluation should evaluate provider current practices, and directly inquire about what is most needed to tailor the E&T to unique populations of providers (e.g., underserved or remote areas).

While collective results are promising, findings highlight the need for additional and targeted study on TMH E&T in terms of defining necessary competencies for providers, greater diversity of study location and demographic considerations, and long-term follow-up to clarify provider ongoing use and outreach activities (e.g., whether TMH E&T fostered a desire for additional outreach efforts than what the provider offered prior to the training). Future studies should seek to remedy noted limitations.

### 4.4 Application of findings

With consideration of both strengths and areas of improvement of current literature, several recommendations for future application of E&T are presented. Most importantly, field experts, training programs, and guiding organizations must work to consolidate literature to identify essential and universal TMH competencies, both knowledge- and skill-based. Additionally, optimal means of conveying such information (e.g., in-person training, web-based self-guided, hybrid approach) is necessary. As part of this training (or following), supervision of TMH usage is vital (46). Supervision of TMH allows for the trainee to receive targeted feedback of both strengths and areas for improvement, allows for advancement of knowledge and skills, and gatekeeps the profession. To track trainee progress, evaluate training program efficacy and effectiveness, and allow for iterative development of the programs, comprehensive and consistent objective outcome measures are required. Utilization of similar measures across studies will allow for cross-site comparison. Targets of evaluation can include satisfaction, as well as the program’s ability to foster greater TMH knowledge, greater TMH skills, and outreach efforts to rural and underserved communities. Additionally, evaluation should consider cost effectiveness of the programs to optimize both training methodologies and costs to create sustainable and scalable programs that can provide a template for TMH education, while allowing the ability to tailor to different regions, states, provinces, territories, or countries to accommodate different languages, cultures, and contexts. Of important note, E&T is not a one-time endeavor. Similar to other aspects of healthcare, continuing education is a lifelong activity. As such, E&T efforts should be designed around different professional developmental stages. More specifically, E&T should be created and adopted by graduate-level training programs to provide novice providers with education and supervision of TMH practices. Such practices can be augmented and supplemented by additional E&T on internships, fellowships, and residencies. Finally, continuing education activities and programs should be tailored to those more senior providers who already have experiences with TMH, but require updates to ensure adherence to ever-changing evidence-based standards and regulations surrounding the use of technology in healthcare services. To formalize training activities, policy makers (e.g., American Psychological Association, American Psychiatric Association, American Medical Association) who guide and/or accredit healthcare training programs, and license regulators (e.g., state licensing boards) should encourage consistent TMH accreditation standards for training and continuing education requirements for maintenance of licensure.

### 4.5 Limitations of the current study

While the current study is believed to address an identified gap in the literature, it is not without limitations that should be considered in the finding’s interpretation. First, the study pulled from five databases. While broad databases were chosen, it is recognized that additional relevant literature may be available in other databases. Similarly, only peer-reviewed manuscripts were considered for inclusion, potentially excluding relevant literature that has yet to be published, or included in non-peer-reviewed outlets (e.g., books). In addition, this review focused on TMH E&T broadly and did not include discussion of other technologies such as AI, a rapidly emerging technology revolutionizing today’s health care. Further, the study aimed to qualitatively describe current field offerings and guide future directions. Nevertheless, it is recognized that future work should consider quantitively defining and analyzing data to further understand current strengths and limitations. Such future study should also seek to address identified field limitations, such as small sample sizes, limited external validity, poor standardization of competencies across studies, and different methodologies that each create challenges for direct comparison.

## 5 Conclusion

Telemental health has rapidly expanded in recent years, subsequently producing a myriad of different training programs, each with unique strengths and limitations. While meaningful progress has been made related to TMH E&T, additional work is required. As the use of TMH is projected to continue (47), it remains prudent for E&T programs to work towards standardization of training foci and methodologies in line with evidence-based literature and developing literature and policies.

## Data availability statement

The original contributions presented in the study are included in the article/Supplementary material, further inquiries can be directed to the corresponding authors.

## Author contributions

QJ: Writing – original draft, Writing – review & editing. YD: Writing – original draft, Writing – review & editing. JP: Writing – original draft, Writing – review & editing. WZ: Writing – original draft, Writing – review & editing. DC: Writing – original draft, Writing – review & editing. JC: Writing – original draft, Writing – review & editing. FL: Writing – original draft, Writing – review & editing.
