# Supplementary material for: Education and training of telemental health providers: a systematic review
Source: Front Public Health. 2024 May 22;12:1385532. doi: 10.3389/fpubh.2024.1385532 (PMC11152158; doi:10.3389/fpubh.2024.1385532)
Supplement: Supplementary file 1 [file Data_Sheet_1.docx]

**Appendix 1. Search Strategy Overview**

Search conducted on May7th, 2023

Keywords: telemental health,TMH, telepsychology, telepsychiatr*, telebehavioral health, TBH, mobile mental health, internet* therapy, training

Boolean Operators: Used AND, OR to combine keywords for broadening or narrowing the search scope.

The search strategy was tailored to different databases to ensure comprehensive retrieval of relevant literature. We searched the following 5 databases and obtained a total of 1617 articles. After removing duplicates (646), we finally remained 1071 articles.

- Search strategy for Pubmed (obtained 330 articles)

("telemental health"[Title/Abstract] OR TMH[Title/Abstract] OR telepsycholog*[Title/Abstract] OR telepsychiatr*[Title/Abstract] OR "telebehavioral health"[Title/Abstract] OR TBH[Title/Abstract] OR "mobile mental health"[Title/Abstract] OR "internet therapy"[Title/Abstract:~3]) AND training[Title/Abstract] Filters: from 2013 – 2023

- Search strategy for Scopus (obtained 296 articles)

TITLE-ABS-KEY ( ( "telemental health" OR tmh OR telepsycholog* OR telepsychiatr* OR "telebehavioral health" OR tbh OR "mobile mental health" OR "internet* therapy" ) AND training ) AND PUBYEAR > 2012 AND PUBYEAR < 2024 AND ( LIMIT-TO ( DOCTYPE , "ar" ) ) AND ( LIMIT-TO ( LANGUAGE , "English" ) )

- Search strategy for Web of Science (obtained 359 articles)

("telemental health" OR TMH OR telepsycholog* OR telepsychiatr* OR “telebehavioral health” OR TBH OR “mobile mental health” OR “internet* therapy” ）AND training (Topic)

- Search strategy for CINAHL (obtained 482 articles)

Search Terms :("telemental health" OR TMH OR telepsycholog* OR telepsychiatr* OR “telebehavioral* health” OR TBH OR “mobile mental health” OR “internet* therapy”）AND training

Search Options

Limiters - Full Text; Published Date: 20130101-20230531; English Language; Peer Reviewed; Research Article

Expanders - Apply related words; Apply equivalent subjects

Search modes - Find all my search terms

- Search strategy for APA PsycArticles (obtained 151 articles)

Search Terms :("telemental health" OR TMH OR telepsycholog* OR telepsychiatr* OR “telebehavioral* health” OR TBH OR “mobile mental health” OR “internet* therapy”）AND training

Search Options

Limiters - Full Text; Year of Publication: 2013-2023; Published Date: 20130101-20230531; Scholarly (Peer Reviewed) Journals

Expanders - Apply related words; Apply equivalent subjects

Search modes - Find all my search terms
